# Liver Transplantation for Isolated Langerhans Cell Histiocytosis in an Adult

**DOI:** 10.1155/crh/1179811

**Published:** 2025-06-25

**Authors:** Ruiyang Huang

**Affiliations:** University of Miami Miller School of Medicine, Miami, Florida, USA

**Keywords:** CD1a+ cell, Langerhans cell histiocytosis, orthotopic liver transplant, sclerosing cholangitis

## Abstract

Langerhans cell histiocytosis (LCH) is a rare disease of proliferation of histiocytic disorder composed of histologically bland Langerhans cells mixed with reactive mononuclear and granulocytic cells, and often accompanied by eosinophils. These cells are characterized by expression of CD1a, S-100 and Langerin proteins. The clinical presentation ranges from indolent to aggressive, depending on the anatomic site involved which can be unifocal, multifocal, unisystemic, or multifocal and multisystemic disease. Cases involving LCH disease and treatment involving the liver are rare, especially in adult patients. Herein, we discuss a case of a 56-year-old male patient who presented with jaundice, acute abdominal pain, and a history of elevated liver function tests assumed to be caused by fatty liver disease. However, a computed tomography (CT) scan revealed a cholangiocarcinoma with associated biliary dilatation and cirrhosis. Pathological examination revealed Langerhans cell involvement. Negative bone marrow biopsy and bone scan indicated that the patient was indeed suffering from unisystemic LCH with isolated liver involvement causing cirrhosis. Patient underwent orthotopic liver transplantation (LT) and has since shown stable liver function without external therapy for 3 years.

## 1. Introduction

Langerhans cell histiocytosis (LCH) is the proliferation and accumulation of CD1a+ dendritic cells, known as Langerhans cells, and it typically manifests in one or more organ systems such as the skin, lung, bone, bone marrow, and liver [1, 2]. Involvement of LCH in the liver is considered “high risk,” as opposed to skin or bone involvement, and typically it manifests clinically with jaundice, fatigue, and pruritus in the early stages, and hepatomegaly, sclerosing cholangitis (SC), and liver failure in the late stages [1, 3]. Most cases occur in children, and it is more common in males than in females. Diagnosis of liver disease caused by LCH involvement is thought to be often unrecognized and unreported, especially amongst adults [1, 3, 4]. Initial diagnosis of LCH usually reports elevated liver function enzymes; however, the most definitive diagnosis can only be reached after liver biopsy. The presence of CD1a+ dendritic cells during pathological examination of liver biopsy samples is indicative of LCH involvement. Prior to the usage of orthotopic liver transplantation (LT) for LCH treatment, a majority (83%) of those suffering from LCH underwent chemotherapy; however, the prognosis of patients in the later stages with SC or cirrhosis was poor [3]. Upon review, only 10 other cases of liver disease caused by LCH involvement in adults have been found where orthotopic LT was pursued as a treatment option. Long-term follow-up of these patients will be indicative of the efficacy of LT as a treatment option for those patients in the late stages of liver disease caused by LCH.

## 2. Case Report

56-year-old male patient with history for hypertension, benign pituitary tumor, and “fatty liver” presented to our hepatology service in June 2018 for evaluation of elevated liver function tests (LFTs) and cirrhosis. He reportedly had a longstanding history of elevated LFTs as far back as 2012. The main abnormal LFTs are alkaline phosphatase elevation with progression up to 800 U/L, which had been assumed that “fatty liver” was the cause in the past. He presented to Emergency Department in January 2019 with acute abdominal pain and acute onset of jaundice. During his hospitalization abdominal magnetic resonance imaging (MRI) showed “mildly prominent intrahepatic ducts and abrupt termination of the common hepatic duct (CHD) with focal abnormal signaling concerning for cholangiocarcinoma/tumor.” A CT examination indicated “cholangiocarcinoma of the proximal to mid common bile duct (CBD) with associated biliary dilatation and metastatic peripancreatic/portal adenopathy, and cirrhosis.” Endoscopic retrograde cholangiopancreatography (ERCP) demonstrated “stricture in mid CBD with saccular dilation immediately above the stricture, and an additional stricture immediately above this saccular dilation, which appeared to be caused by extrinsic compression with lymph node.” The lymph node biopsy was negative for malignancy.

### 2.1. Diagnosis

Patient underwent exploratory laparotomy, cholecystectomy, and hepaticojejunostomy. Pathology examination showed the extrahepatic bile duct with involvement by LCH, marked chronic inflammation and fibrosis with mucosal ulceration. The wedge liver biopsy showed involvement by LCH, biliary fibrosis, cholestasis, and advanced fibrosis. Gallbladder demonstrated chronic cholecystitis.

Subsequent bone marrow biopsy was negative for LCH. Current recommendations published in 2022 suggest FDG-PET/CT for LCH patients to determine tumor staging [5], however this was not done in our patient. Instead, bone scan did not show any osseous involvement by LCH. Patient was diagnosed as LCH with isolated liver involvement and decompensated cirrhosis.

### 2.2. Treatment

**Figure 1:**
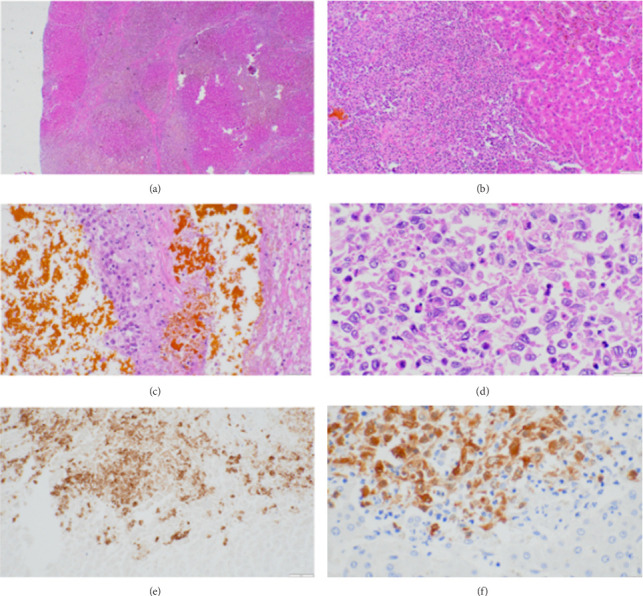
(a) Liver showing biliary-type cirrhosis with severe cholestasis (scale bar = 500 μm, 2X). (b) Expended portal area showing mixed inflammatory infiltrate and one destructed interlobular bile duct (scale bar = 100 μm, 10X). (c) Secondary sclerosing cholangitis with lithiasis and sheets of Langerhans cells infiltrate (scale bar = 50 μm, 20X). (d) High-power view showing the Langerhans cells are large histocytes with oval irregular folded nuclei, vesicular chromatin, inconspicuous nucleoli, and moderately abundant eosinophilic cytoplasm (scale bar = 20 μm, 40X). (e) Immunostain showing the Langerhans cells are positive for CD1a) (scale bar = 50 μm, 20X). (f) Immunostain shows the Langerhans cells are positive for S100 (scale bar = 20 μm, 40X).

Although Next Generation Sequencing (NGS) and targeted therapies are emerging treatments of histiocytic neoplasms, particularly for identifying and addressing MAPK pathway mutations [5], neither was considered or pursued in this case due to the patient's urgent clinical status and the immediate need for LT. LT was performed in July 2019. Pathology examination of the explanted liver showed LCH involvement with severe cholestasis and biliary type cirrhosis (Figure 1(a)). Portal areas showed mixed inflammation including lymphocyst, eosinophils, and Langerhans histiocytes (Figure 1(b)), bile ductular reaction, and severe SC with Langerhans histiocyte infiltrate with hepatolithiasis (Figure 1(c)). The Langerhans histiocytes demonstrated oval irregular folded nuclei, vesicular chromatin, inconspicuous nucleoli and ill defined, finely granular or vacuolated eosinophilic cytoplasm (Figure 1(d)), which were positive for S100 (Figure 1(e)) and CD1a (Figure 1(f)). No BRAF mutation was detected by molecular tests in this case.

### 2.3. Outcomes

The patient has been undergoing surveillance without any therapy for LCH. His liver function has been stable for 3.5 years with no notable adverse and unanticipated events relating to his liver transplant.

## 3. Discussion

Although liver involvements in LCH cases are rare, reported as 10.1%–18% in pediatric patients with LCH, and cases of hepatic involvement in adults leading to SC are rarely reported and reviewed [2]. Disease prognosis is significantly worse with liver involvement; a fatality rate of 30%–50% is observed versus less than 10% when the liver is not involved [1, 3].

Diagnosis of this disease is also difficult without liver biopsy. LCH shares common clinical and pathological symptoms with many liver diseases such as jaundice, cholangitis, cirrhosis, pruritus, fatigue, and elevated LFTs. In our case, our patient had presented a year earlier with elevated liver function enzymes and it was assumed that these were only the result of his fatty liver disease. Only after follow-up and the presence of later stage symptoms such as jaundice and SC was a definitive diagnosis of LCH reached after immunohistochemical analysis proved the presence of CD1a+ Langerhans cells in the patient's liver biopsy.

As for treatment of unisystemic LCH in the liver or spleen, systemic therapies including cladribine, cytarabine, or vinblastine and prednisone are recommended while smoking cessation is the first line recommendation for unisystemic pulmonary LCH [5]. However, LT has shown success specifically in treating LCH involving end-stage liver disease involving cirrhosis [6–8].

Ziogas et al. [4] reviewed LT cases from 1987 to 2018 in a US population-based analysis containing a total of 60 patients with secondary SC due to LCH undergoing LT. The median age at LT was 3.0 years of age. LT recipients' survival outcomes were acceptable and the disease recurrence rate to the liver was low. The conclusion was that LT should be considered in patients with liver failure secondary to LCH even in the setting of active multisystem disease, if recipients are expected to have good survival and options for salvage therapy in the setting of disease reactivation. Of note, only 5 patients were adults, and the clinical presentation of these cases was not documented in this review. To the best of our knowledge, there were only 10 adult LCH patients treated with LT from 1990 to present. The clinical features, prognosis, and treatment are summarized in Table 1.

As seen in Table 1, even among this handful of cases lie differences in patient symptoms, treatment methods, and treatment outcomes. For example, the current standard of care for multisystemic LCH includes systemic chemotherapy (vinblastine, prednisone, and mercaptopurine) [15, 16], whereas the standard for unifocal LCH has been surgical resection [17]. Analysis of long-term outcomes of LCH showed a 5-year overall survival (OS) rate of 88% with these first-line treatments; however, when a “high risk organ,” such as the liver or spleen, was involved, their OS rate worsened (Hazard Ratio, 10.8; 95% CI, 3.3–35.5; *p* < 0.001), and patients were 10.8 times more likely to not have survived at 5 years [17]. Interestingly, in two of the cases reviewed, chemotherapeutics either worsened the severity of the patient's liver injury [9] or did not have any effect on the patient's liver damage [8]. Whereas in another case, recurrence of LCH was successfully controlled using chemotherapy [11]. Fortunately, a similarity found among these cases is the scarcity of LCH recurrence at follow-up. Among these cases, only three have recorded follow-ups with a longer post-operation duration than our patient. Our current case adds to the small list of LCH patients who have undergone LT in adults, and long-term follow-up of these patients will be the key to understanding the extended efficacy of transplantation as a treatment for LCH-related liver disease.

### 3.1. Learning Points

Although rare, LCH involvement in the liver is a serious concern for pediatric and adult patients with LCH alike, as its involvement in the liver greatly increases the fatality rate of LCH. Diagnosis of LCH is complicated by similarities in clinical symptoms with many other liver diseases such as fatty liver disease or primary SC. The main differentiator between LCH and other liver diseases that also result in end-stage liver disease is the presence of CD1a+ dendritic cells, especially proximal to the bile ducts. Orthotopic LT has emerged as a possible treatment option, especially for patients in decompensated cirrhosis secondary to liver involvement. There are very few other patients that have been monitored for a longer duration than our patient after LT for LCH liver disease, and the relative absence of recurrence of liver disease in these patients is a promising sign of the efficacy of LT as a treatment for LCH related liver disease.

## Figures and Tables

**Table 1:** LCH cases treated with liver transplantation.

Case	Source, year	Sex	Age (diagnosis)	Age (liver transplant)	Clinical presentation	Complications	Follow-up
1.	Our case	M	56	57	Jaundice, sclerosing cholangitis and cirrhosis	None	42 months
2.	Tang, et al. 2017	M	31	39	Fatigue, anorexia, jaundice, pruritus, hepatic lesions	None	54 months no evidence of recurrence [9]
3.	Scanzi, et al. 2015	M	49	49	Cirrhosis, purpuric scalp lesions, cholestasis.	Died on postoperative day 17 from invasive aspergillosis	Died on postoperative day 17 from invasive aspergillosis [7]
4.	Lee, et al. 2011	M	39	42	Gallstone pancreatitis, pruritus, scapula mass,	None	2 years and normalized liver function [6]
5.	Griffiths, et al. 2006	F	55	64	Jaundice, pruritus, sclerosing cholangitis, portal to portal fibrosis, peritoneal nodules	Right lobe split liver transplant, hepatic artery thrombosis on the post-operative day 4. Re-liver transplant	Normal liver function after 14 months following second liver transplant
6.	Caputo, et al. 2004	F	41	43	Abdominal lesions, scalp lesions, hepatosplenomegaly, polyuria, polydipsia, osteolytic lesions	None	163 months no evidence of recurrent hepatic disease [10]
7.	De Diego, et al. 2002	M	24	28	Jaundice, hepatosplenomegaly, sclerosing cholangitis	Moderate acute rejection on post-operative day 10	48 months following liver transplant presents with diabetes, hypothyroidism, LCH reactivation, this time controlled with chemotherapy [11]
8.	Sampathkumar, et al. 2002	F	29	34	Polyuria, polydipsia, fatigue, headache, amenorrhea, hepatic lesions, sclerosing cholangitis, hyperthyroidism	Goiter formed 3 months before transplant, controlled with chemotherapy [12]	Not available
9.	Rice, et al. 2000	M	34		Abdominal pain, liver nodule	None	48 months no evidence of LCH [13]
10.	Rice, et al. 2000	F	39	39	Cirrhosis, liver nodules	None	6 months no evidence of LCH [13]
11.	Stieber, et al. 1990	M	Not available	Not available	Not available	Not available	Died 14 months post-transplant from pulmonary embolism, no LCH recurrence [14]

## Data Availability

Data sharing is not applicable to this article as no new data were created or analyzed in this study.

## References

[1] Fu Z., Li H., Arslan M. E., Ells P. F., Lee H. (2021). Hepatic Langerhans Cell Histiocytosis: A Review. *World Journal of Clinical Oncology*.

[2] Hatemi I., Baysal B., Senturk H., Behzatoglu K., Bozkurt E. R., Ozbay G. (2010). Adult Langerhans Cell Histiocytosis and Sclerosing Cholangitis: A Case Report and Review of the Literature. *Hepatology International*.

[3] Abdallah M., Généreau T., Donadieu J. (2011). Langerhans’ Cell Histiocytosis of the Liver in Adults. *Clinics and Research in Hepatology and Gastroenterology*.

[4] Ziogas I. A., Kakos C. D., Wu W. K. (2021). Liver Transplantation for Langerhans Cell Histiocytosis: A US Population‐Based Analysis and Systematic Review of the Literature. *Liver Transplantation*.

[5] Goyal G., Tazi A., Go R. S. (2022). International Expert Consensus Recommendations for the Diagnosis and Treatment of Langerhans Cell Histiocytosis in Adults. *Blood*.

[6] Griffiths W., Davies S., Gibbs P., Thillainayagam A., Alexander G. (2006). Liver Transplantation in an Adult With Sclerosing Cholangitis Due to Langerhans Cell Histiocytosis. *Journal of Hepatology*.

[7] Lee R. J., Leung C., Lim E. J. (2011). Liver Transplantation in an Adult With Sclerosing Cholangitis Due to Multisystem Langerhans Cell Histiocytosis. *American Journal of Transplantation*.

[8] Scanzi J., Goutte M., Teilhet C., Abergel A. (2015). When Should We Consider Transplantation in Adult Patients With Sclerosing Cholangitis Due to Multisystem Langerhans’ Cell Histiocytosis?. *Digestive and Liver Disease*.

[9] Tang Y., Zhang Z., Chen M. (2017). Severe Sclerosing Cholangitis after Langerhans Cell Histiocytosis Treated by Liver Transplantation. *Medicine*.

[10] Caputo R., Marzano A. v., Passoni E., Fassati L. R., Agnelli F. (2004). Sclerosing Cholangitis and Liver Transplantation in Langerhans Cell Histiocytosis: A 14-Year Follow-Up. *Dermatology*.

[11] de Diego A., Escudero M., Catalina M. V. (2002). Recurrence of Langerhans Cell Histiocytosis in the Graft After Liver Transplantation in Adults. *Transplantation Proceedings*.

[12] Sampathkumar S., Younger C., Cramer H., Chalasani N., Skierczynski P. A. (2002). Langerhans’ Cell Histiocytosis Involving the Pituitary, Thyroid, Lung, and Liver. *Endocrine Practice*.

[13] Rice A. J., Wyatt J. I. (2000). Solitary Langerhans Cell Histiocytosis in Association With Primary Biliary Cirrhosis. *Histopathology*.

[14] Stieber A. C., Sever C., Starzl T. E. (1990). Liver Transplantation in Patients With Langerhans’ Cell Histiocytosis. *Transplantation*.

[15] Rodriguez-Galindo C., Allen C. E. (2020). Langerhans Cell Histiocytosis. *Blood*.

[16] Henter J. I., Tondini C., Pritchard J. (2004). Histiocyte Disorders. *Critical Reviews In Oncology-Hematology*.

[17] Goyal G., Acosta-Medina A. A., Abeykoon J. P. (2023). Long-Term Outcomes Among Adults With Langerhans Cell Histiocytosis. *Blood Advances*.

